# Study on the Performance of Liquid-Solid Contact Resistance Based on Magnetohydrodynamic Micro-Angular Vibration Sensor

**DOI:** 10.3390/s22239204

**Published:** 2022-11-26

**Authors:** Ganmin Xia, Weixiao Tuo, Xingfei Li, Xinyu Liu

**Affiliations:** 1 State Key Laboratory of Precision Measuring Technology and Instruments, Tianjin University, Tianjin 300072, China; 2 Taihu Laboratory of Deepsea Technological Science, Wuxi 214000, China

**Keywords:** liquid-solid contact resistance, conductive fluid, fluid ring, dynamic contact resistance

## Abstract

In this paper, the influence of the contact resistance between the conductive fluid and the metal electrode on the output characteristics of the magnetic fluid micro-angular vibration sensor (MHD sensor) is theoretically analyzed. The contact resistance models based on the solid-solid electric contact theory are established based on the resistivity, temperature, pressure and angular vibration of the materials between the conductive fluid and the metal electrode. The contact resistance was tested by setting up an experimental platform and making conductive fluid rings with electrode materials of Ag, Cu and Ti. The results show that the static contact resistance between the conductive fluid and the metal electrode is positively correlated with the material resistivity and temperature, and negatively correlated with the surface roughness and contact pressure of the metal electrode. The dynamic contact resistance fluctuation is proportional to the amplitude of the input voltage of the angle shaker and inversely proportional to the square of the input frequency. At the same time, reducing contact resistance can improve the MHD sensor’s performance.

## 1. Introduction

A magnetohydrodynamic micro-angle vibration sensor (MHD sensor) is a kind of measuring high frequency, lower value angular vibration sensor, with wide band, lower noise, impact and characteristics of small volume, and it is widely used in high-precision deep space spacecraft, such as relay satellite mirror RME [1], land surface observation satellite ALOS [2], environmental satellite GOES-N [3], etc.

The fluid ring is the core sensitive element of MHD sensors [4,5], which is filled with conductive fluid [6,7], and the upper and lower electrodes of the fluid ring are composed of high conductivity materials. The upper and lower electrodes and the conductive fluid form a closed loop with the primary of the coil amplifier. The conductive fluid and the electrode form liquid-solid contact at the contact interface, resulting in contact resistance (fluid ring contact resistance). Contact resistance is an additional resistance of two contact surfaces at the contact site due to roughness and the film layer, which is an electrical contact phenomenon. Fluid ring contact resistance is an additional resistance at the contact surface due to the influence of the viscosity and surface tension of the conductive fluid, which cannot completely wet the surface of the rough metal electrode. When the MHD sensor generates the induced electromotive force in the external vibration environment, current will be generated in the closed loop. Due to the existence of contact resistance, voltage noise will be generated between the contact interfaces. The voltage noise will be superimposed in the output signal of the induced potential, reducing the signal-to-noise ratio of the MHD sensor and affecting the sensitivity and bandwidth.

Most of the research on contact resistance has focused on solid-solid contact resistance, and there has been less research on liquid-solid contact resistance. Indeed, ref. [8] proposed that the contact between two solid surfaces is actually completed by a series of concave and convex microscopic spots, and the total area of spots is the actual contact area of the contact between two rough surfaces, which is far smaller than the apparent contact area. Studies in [9,10] proposed a three-dimensional finite element model of contact resistance, and explored the constants in the model through static contact resistance experiment, provided the relationship between contact resistivity and pressure, material hardness and the average resistivity of experimental materials and obtained the relationship between contact pressure and contact resistance. For the contact resistance affected by vibration, fretting is considered to be one of the main factors affecting the electrical connection [11], a small amplitude relative motion between two contact surfaces causes discontinuity of the electrical contact material, contact wear and corrosion, resulting in the change in the contact resistance [12]. The local relative motion can be used to represent the contact resistance [13]. Indeed, ref. [14] showed that the change of connector contact resistance under vibration is mainly due to the periodic change in contact area caused by the relative motion of the contact interface.

This paper studies the influential factors of liquid-solid contact resistance of a fluid ring based on the solid-solid electric contact theory, and verifies the influence of different electrode materials on liquid-solid contact resistance through simulation and experimentation. The paper is organized as follows: Section 2 introduces the working principle and contact resistance model of the MHD sensor. In Section 3, theoretical models of liquid-solid contact resistance based on conductive fluid ring are established by using the principle of electric contact. Section 4 sets up the fluid ring contact resistance experimental platform. Section 5 analyzes the test results, and Section 6 is the conclusion.

## 2. MHD Sensor Working Principle and Output Model

### 2.1. MHD Sensor Working Principle

**Figure 1:**
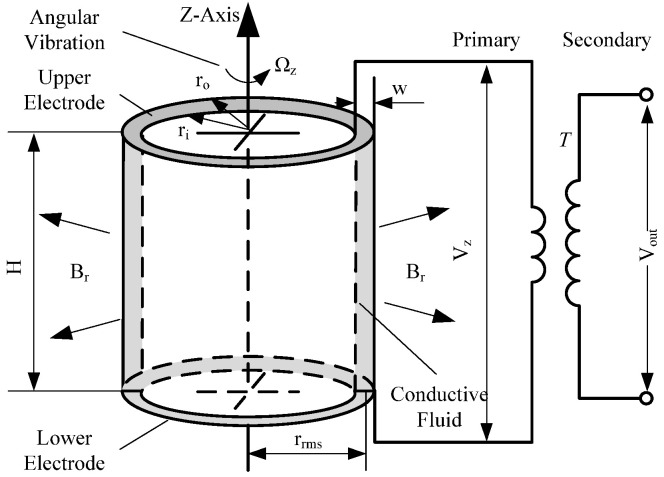
Working principle of the MHD sensor.

The working principle of the MHD sensor is based on Faraday’s law of electromagnetic induction. As shown in Figure 1, the fluid ring is composed of conductive channels on the upper and lower walls, and insulated is on the inner and outer walls. The channel is filled with conductive fluids. The radially radiated magnetic field generated by the permanent magnet passes vertically through the conductive fluid along the diameter of the fluid ring. The fluid ring is connected to the permanent magnet and its shell. When the angular vibration *Ω_Z_* is generated relative to the sensitive axis Z of the MHD sensor, due to the large inertia of the conductive fluid and the static relative to the shell, the relative motion between the conductive fluid and the permanent magnet magnetic field is generated, and the magnetic field line is cut, and the potential difference *V_z_* is generated between the upper and lower conductive surfaces. The potential difference *V_z_* is proportional to the input angular vibration *Ω_Z_*, and the *V_out_* can be detected by the coil amplifier and subsequent circuit amplification processing. The relevant information of the angular vibration can be solved by analyzing the collected signal.

### 2.2. MHD Sensor Output Model

According to Faraday’s law of electromagnetic induction and the principle of magnetohydrodynamics, the fluid ring model can be expressed as:

(1) $$\frac{V_{z}\left(s\right)}{\Omega_{z}\left(s\right)}=\frac{B_{r}r_{rms}Hs}{s+\frac{\mu_{f}}{\rho w^{2}}\left(1+Ha^{2}\right)}$$

where *B_r_* is the magnetic induction intensity at the conductive fluid; H is fluid ring height; *r_rms_* is the root mean square radius of the fluid ring. *ρ* is the density of conductive fluid; w is the width of the fluid ring, *w* = *r_o_-r_i_*, *r_o_* and *r_i_* are the outer radius and inner radius of the fluid ring, respectively; *Ha* is Hartmann constant; *μ_f_* is the kinematic viscosity of conductive fluid.

**Figure 2:**
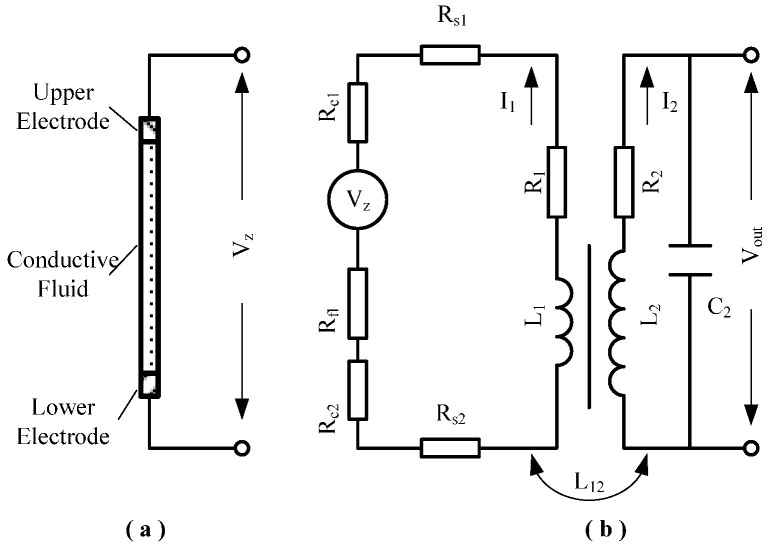
Schematic diagram of fluid loop section (**a**) and coil amplifier equivalent circuit (**b**).

The detection of *V_z_* of the fluid ring output signal of high frequency weak signal is one of the key problems of the MHD sensor. In order to obtain the higher amplification ratio as far as possible, it is also necessary to consider the bandwidth. Simple circuit amplification cannot meet the requirements, so the signal amplification process uses a passive device coil amplifier for primary amplification to suppress noise. Then comes the second stage of amplification and filtering, and other post-processing. The section diagram of the fluid ring cavity is shown in Figure 1 of the MHD sensor, and the equivalent circuit diagram of the coil amplifier is shown in Figure 2. In the figure, the output-induced electromotive force *V_z_* of the fluid ring is equivalent to a voltage source, the resistance of the conductive fluid and the metal electrode and their contact resistance, and the primary composition loop of the coil amplifier. Due to the small number of primary turns of the coil amplifier, eddy current loss resistance and parasitic capacitance are not considered; only the copper loss of the primary coil is considered, which is equivalent to the series of resistance and inductor. Due to the large number of turns of the secondary coil, copper loss of the secondary coil and parasitic capacitance between the windings of the secondary coil should be considered. Therefore, the secondary coil is equivalent to a series of resistance and inductor, and then a parallel connection with the capacitor. Then, the equivalent resistance of the coil amplifier primary loop can be expressed as:

(2) $$R=R_{s1}+R_{c1}+R_{fl}+R_{c2}+R_{s2}+R_{1}$$

where *R* is the total resistance of the primary coil; *R_s_*_1_ is upper electrode resistance; *R_s_*_2_ is the lower electrode resistance; *R_fl_* is conductive fluid resistance; *R_c_*_1_ is the contact resistance between the conductive fluid and the upper electrode; *R_c_*_2_ is the contact resistance between the conductive fluid and the lower electrode; *R*_1_ is the coil amplifier resistance.

According to Kirchhoff’s law, the equivalent circuit equation of the coil amplifier is:

(3) $$\{\begin{array}{l} RI_{1}+sL_{1}I_{1}-sL_{12}I_{2}=V_{Z}\\ R_{2}I_{2}+\frac{I_{2}}{sC_{2}}+sL_{2}I_{2}-sL_{12}I_{1}=0 \end{array}$$

It can be obtained from Equation (3):

(4) $$\{\begin{array}{l} I_{1}=\frac{V_{Z}+sL_{12}I_{2}}{R+sL_{1}}\\ I_{2}=\frac{V_{Z}sL_{12}}{R+sL_{1}}\frac{1}{R_{2}+\frac{1}{sC_{2}}+sL_{2}-\frac{s^{2}L_{12}^{2}}{R+sL_{1}}} \end{array}$$

Therefore, the output voltage *V_out_* can be obtained:

(5) $$V_{out}\left(s\right)=I_{2}\frac{1}{sC_{2}}$$

Thus, the transfer function between the input and output of the coil amplifier is obtained as follows:

(6) $$\begin{array}{l} \frac{V_{out}\left(s\right)}{V_{Z}\left(s\right)}=\frac{sL_{12}}{R+sL_{1}}\frac{1}{R_{2}+\frac{1}{sC_{2}}+sL_{2}-\frac{s^{2}L_{12}^{2}}{R+sL_{1}}}\\ =\frac{1}{\frac{C_{2}\left(L_{1}L_{2}-L_{12}^{2}\right)}{R}s^{3}+\frac{C_{2}\left(L_{1}R_{2}+L_{2}R\right)}{R}s^{2}+\frac{\left(C_{2}RR_{2}+L_{1}\right)}{R}s+1}\frac{L_{1}s}{R} \end{array}$$

The noise source of the MHD sensor mainly includes external noise and internal noise. Due to the imperfect electromagnetic shielding of the sensor, the external electromagnetic interference will affect the output of the sensor, and the impedance of the equivalent electrical components inside the sensor is the main source of internal noise. The MHD sensor’s internal resistance thermal noise voltage noise spectral density *E_s_* can be calculated according to the resistance thermal noise spectral density formula:

(7) $$E_{s}=\sqrt{4\kappa TRN^{2}}$$

where *κ* = 1.38 × 10^−23^ J/K is Boltzmann’s constant; *T* is absolute temperature (K); *N* is the amplification ratio of the coil amplifier.

According to Equations (6) and (7), the smaller the primary resistance R is, the smaller the impact on the output of the MHD sensor will be, and the more stable the coil amplifier system will be. In Equation (2), the primary resistance is the sum of the resistance values of all materials in the primary loop and the contact resistance values between adjacent materials. In addition to the contact resistances *R_c_*_1_ and *R_c_*_2_, the other resistance values are all composed of the resistance values of the metal materials themselves. When the structure of the fluid ring is determined, the resistance value is fixed. There are many factors, such as conductivity, temperature, pressure, etc., affecting the contact resistance *R_c_*_1_ and *R_c_*_2_. Therefore, reducing the contact resistance value is beneficial to reduce the primary resistance *R*.

## 3. Fluid Ring Contact Resistance Model

Similar to the solid-solid contact resistance, the factors affecting the liquid-solid contact resistance of the fluid ring mainly include resistivity and wettability of conductive fluid and metal electrode, contact area, surface roughness, contact pressure, vibration, etc.

(1) Material conductivity: According to the Holm contact resistance model [15], the contact resistance between conductive fluid and metal electrode can be expressed as:

(8) $$R_{c}=\frac{\rho_{1}+\rho_{2}}{4\sum_{i=1}^{n}r_{i}}$$

where *R_c_* is the contact resistance; *ρ*_1_ and *ρ*_2_ are the resistivity of conductive fluid and metal material electrode; *n* is the number of conductive spots on the contact surface between conductive fluid and metal electrode; *r_i_* is the equivalent radius of conductive spots. According to Equation (8), the resistivity has a great influence on the contact resistance. A small resistivity can not only reduce the contact resistance, but also help to reduce the resistance of the metal electrode. The greater the number of conductive spots, the smaller the contact resistance. The larger the surface roughness of the metal electrode, the more discontinuous contact points, the fewer conductive spots, the smaller the ratio of the actual contact area to the apparent contact area, and the higher the contact resistance.

(2) Temperature: The resistivity of conductive materials changes with temperature [16,17], which can be expressed as follows:

(9) $$\{\begin{array}{c} \rho_{sT}=\rho_{s0}\left(1+\gamma_{S}T\right)\\ \rho_{lT}=\rho_{l0}\left(1+\gamma_{l}T\right) \end{array}$$

where *ρ_sT_* and *ρ_lT_* are the resistivity of the metal electrode and conductive fluid at temperature *T* °C; *ρ_S_*_0_, *ρ_l_*_0_ are the resistivity of the metal electrode and conductive fluid at 0 °C; *γ_S_* and *γ_l_* are the resistivity temperature coefficients of the metal electrode and conductive fluid.

Therefore, substitute Equation (9) into Equation (8) to obtain:

(10) $$\begin{array}{c} R_{ct}=\frac{\rho_{s0}\left(1+\gamma_{S}T\right)+\rho_{l0}\left(1+\gamma_{l}T\right)}{4\sum_{i=1}^{n}r_{i}}\\ =R_{c0}+\frac{\rho_{s0}\gamma_{S}+\rho_{l0}\gamma_{l}}{4\sum_{i=1}^{n}r_{i}}T \end{array}$$

In Equation (10), with the increase in temperature, the resistivity of conductive fluid and metal electrode increases, and the contact resistance accordingly increases.

**Figure 3:**
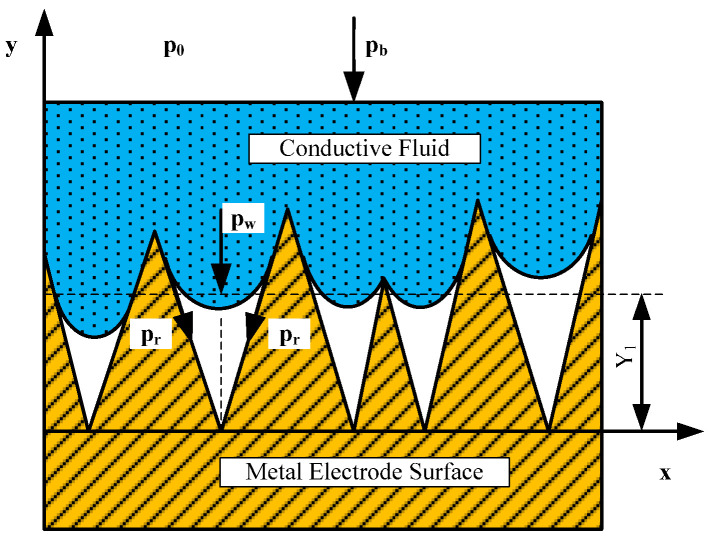
Schematic diagram of contact morphology between conductive fluid and rough surface.

(3) Pressure: When the conductive fluid wets the rough surface of the metal electrode, according to the Cassie-Baxter model [18], the contact morphology between the conductive fluid and the metal electrode is shown in Figure 3. When the conductive fluid touches the metal electrode conical convex point, its wetting notch penetrates to deep *Y*_1_, *Y*_1_, which is called the wetting depth [19,20]. Only the conical bulge above *Y*_1_ will actually make contact with the conductive fluid. When the current passes through the contact interface between the conductive fluid and the metal electrode, the contact resistance is related to the shape of the conical convex point.

As shown in Figure 3, assuming that the gas in the micro-cavity is an ideal gas, the wetting depth *Y*_1_ can be expressed as:

(11) $$Y_{1}\approx \sigma \left(\sqrt{\frac{2\sigma^{\prime }}{\pi }}\cdot \sqrt[{3}]{\frac{2p_{0}}{p_{b}+p_{w}+p_{r}}}\right)$$

where *p_b_* is the pressure applied on the conductive fluid; *p_w_* is the liquid column pressure generated by the conductive fluid on the microcavity. *p_r_* is the capillary pressure at the interface between electrode surface and conductive fluid. *σ*′ is the standard deviation of surface roughness height.

According to Equation (11), as the external pressure *P_b_* increases, the average height of the microcavity *Y*_1_ decreases, the depth of the conductive fluid wetting the surface of the metal electrode increases [21], the actual contact area between the metal electrode and the conductive fluid increases, and the contact resistance decreases.

(4) Angular vibration excitation: Under the angular vibration excitation, the conductive fluid and the metal electrode have relative motion, which causes the change in contact area and the change in contact resistance [22,23,24,25,26]. Angular vibration is set to $\Omega_{z}=KU_{r}\sin\left(2\pi f_{r}t\right)$, *U_r_* and *f_r_*, respectively. Angle vibration excitation, input voltage amplitude and frequency *K* for the parameters are associated with the high frequency vibration table. The relationship between the output angular vibration of the high frequency shaking table and the input voltage can be expressed as follows:

(12) $$U_{r}=K_{1}\cdot a=K_{1}\cdot \theta \cdot {\left(2\pi f_{r}\right)}^{2}$$

where *K*_1_ is the constant related to the high frequency shaking table; *a* is angular acceleration; *θ* is the angle position.

**Figure 4:**
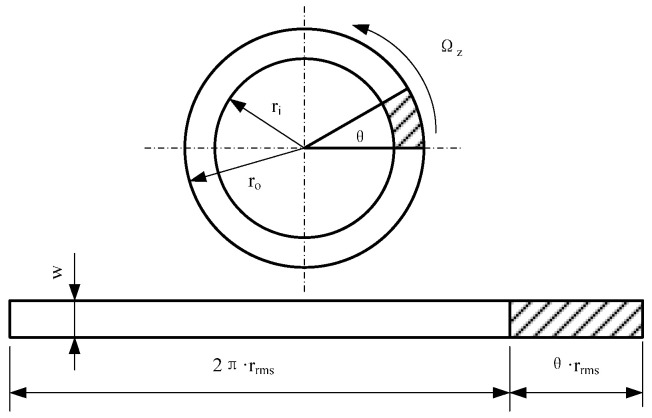
Variation of contact area between conductive fluid and metal electrode under angular vibration.

Therefore, in the process of angular vibration excitation, the fluctuation of fluid ring contact resistance can be expressed as:

(13) ΔRc=(ρ1+ρ2)HS+ΔS−(ρ1+ρ2)HS=RcS·ΔS

where *R_c_* is the contact resistance; *S* is the static contact area between the conductive fluid and the metal electrode; Δ*S* is the change of contact area during angular vibration, as shown in Figure 4, then:

(14) $$\Delta S=\theta \cdot w\cdot r_{rms}$$

The fluid ring contact resistance fluctuation can be expressed as:

(15) $$\Delta R_{c}=K_{2}U_{r}/f_{r}^{2}$$

where *K*_2_ is the parameter related to angular vibration. According to Equation (15), when the fluid ring is excited by the high-frequency shaking table, the fluctuation of the contact resistance of the fluid ring is proportional to the amplitude of the angular vibration voltage and inversely proportional to the square of the angular vibration frequency.

## 4. Experimental Setup

### 4.1. Experimental Platform

**Figure 5:**
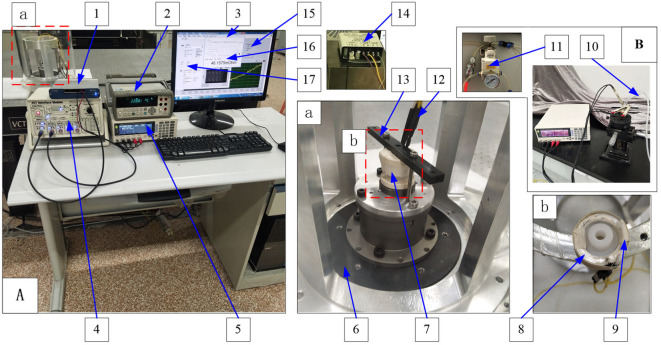
The experimental platform. A—Static temperature experiment, different material experiment and dynamic experiment; B—Static pressure experiment. 1—Acquisition card; 2—Temperature monitor; 3—Industrial computer; 4—High frequency shaking table controller; 5—Resistance meter; 6—High frequency shaking table; 7—Heating device; 8—Fluid ring; 9—Temperature sensor; 10—Silica gel tube; 11—Pressure regulating valve; 12—Leading wire; 13—Installation tooling; 14—Temperature control module; 15—Temperature control and monitoring procedures; 16—Contact resistance acquisition program; 17—High frequency shake table control program.

The contact resistance test platform was composed of hardware and software. The hardware mainly included fluid ring 8, high-frequency shaking table 6, resistance meter 5, acquisition card 1, temperature control circuit 14, air source and pressure regulating valve 11, etc. Software mainly included temperature control and monitoring program 15, contact resistance acquisition program 16, high-frequency shaking table control program 17, etc., as shown in Figure 5.

Fluid ring 8 was the experimental object of contact resistance, and its upper and lower electrodes were made of copper (5.7 × 10^7^ S/m), silver (6.3 × 10^7^ S/m) and titanium (2.3 × 10^6^ S/m), respectively, with high conductivity and no magnetism for the MHD working principle. Due to the soft texture of pure silver, a copper electrode was used for overall silver plating, so that the electrode surface and conductive fluid contact material were silver. Four groups of fluid rings were made for the contact resistance test. The upper and the lower electrode materials were 1#: Ag-Ag, 2#: Cu-Ag, 3#: Cu-Cu and 4#: Ti-Ti, respectively. The fluid rings were cleaned with petroleum ether and acetone before assembly and bonded with epoxy resin (Phoenix epoxy resin, Nantong Xingchen synthetic material Co. Ltd., Nantong, China). The copper electrode lead wire was welded with the upper electrode and the lower electrode by lead-free solder paste (Huaqing Solder M&T Co., Shanghai, China) to draw out the contact resistance for convenient measurement.

The high-frequency shaking table 6 used 105-AVT-I/M from ACUTRONIC, Switzerland, and could provide a stable angular vibration output from 25 Hz to 2.5 kHz. Acquisition card 1 adopted the NI PCI-6289 board from NI Company, United States, which had a 24-bit A/D acquisition card, 32 analog inputs and 4 analog outputs. Resistance meter 5 used RM3542-01 from HIOKI, Japan, through the DC four terminal method to test the contact resistance, measurement range: 0 Ω (10^−7^ Ω)~120 MΩ, and could achieve a high resolution 7-bit display. The air source was dry compressed air and the maximum atmospheric pressure was 0.85 MPa. The pressure regulating valve adopted was a SMC precision pressure regulating valve; range was 0.6 MPa, accuracy was 0.002 MPa. The temperature control circuit adopted was a temperature control module TCM-X107 (Shenzhen Yuxin Electronics Co., LTD, Shenzhen, China), and control accuracy was ±0.01 °C.

Contact resistance acquisition program 16 was used to record the contact resistance of the fluid ring. The recorded resistance value was written into a CSV format file, which can be used to adjust the acquisition time interval of contact resistance. The high-frequency shaking table control program 17 was written using LabVIEW software to control the angular vibration input voltage amplitude and frequency of the high-frequency shaking table.

### 4.2. Experimental Method

The static experiment of contact resistance of fluid ring included a static pressure experiment and static temperature experiment, static and dynamic experiments of electrodes of different metal materials and a static noise experiment of the MHD sensor with the fluid ring with electrodes of different metal materials.

(1) Static pressure experiment: the contact resistance of the fluid ring is measured by the DC four-terminal method. The two Kelvin clips of the resistance meter RM3542-01 are respectively clamped to the leading wire of the upper electrode and the lower electrode of the fluid ring, and the air pressure is applied to the conductive fluid through the filling port. Revision: The electrode material of the conductive fluid ring used in the experiment is 3#: Cu-Cu, in which the conductive fluid is carried out by automatic vacuum perfusion and atmospheric pressure manual perfusion. The ambient temperature was 25 °C ± 1 °C.

(2) Static temperature experiment: a PI thin film resistance heater is wrapped on the cylindrical surface outside the fluid ring cavity, and the thin film Pt resistance CRZ-1632 was pasted on the metal electrode. The temperature of the conductive fluid in the fluid ring is controlled by the TCM-X107 temperature control module, and the static resistance of the fluid ring at different temperatures is measured using the four-wire resistance experiment method.

(3) Static experiment of electrodes of different metal materials: the fluid ring is fixed on the 105-AVT-I/M high-frequency vibration table by special tooling, as shown by No. 13 in Figure 5. The mounting tool is screwed on to the shaking table, and the fluid ring is tightened to the mounting tool. Before the experiment, the leading wire of the sensor electrode is lengthened with a thick wire (the resistance value is 4.3 mΩ before it is lengthened, and 45.78 mΩ after it is lengthened.). This is connected to the input end of the resistance meter HIOKI RM3542-01 through the Kelvin clip and wrapped with tape. At the same time, the wire and the Kelvin clip are fixed to the high frequency shaking table body to avoid the influence of high frequency vibration on the contact between the wire and the resistance meter, to ensure that in the experiment loop, in addition to the relative movement between the conductive fluid and the electrode, the other parts are kept relatively static and do not affect the experiment results. The experiment is carried out in a constant temperature environment, ignoring the influence of wires and other environmental temperature fluctuations. The CRZ-1632 thin film Pt resistance is pressed against the outer surface of the lower electrode of the fluid ring, making full contact, and fixed with tape. The temperature corresponding to the Pt resistance of the film and the contact resistance of the fluid ring are monitored and recorded using the four-terminal method.

(4) Dynamic experiment: input the specified amplitude and frequency through the LabView program to control the output angle vibration of the high-frequency shaking table. Meanwhile, the fluid ring moves with the high-frequency shaking table, and the contact resistance and corresponding temperature changes of the fluid ring in the vibration process are recorded.

(5) Static noise and frequency response experiment: different metal electrodes are assembled into the MHD sensors, except the fluid ring, the rest of the parts are the same as the static noise and frequency response experiment of the MHD sensor.

## 5. Experimental Results and Analysis

**Figure 6:**
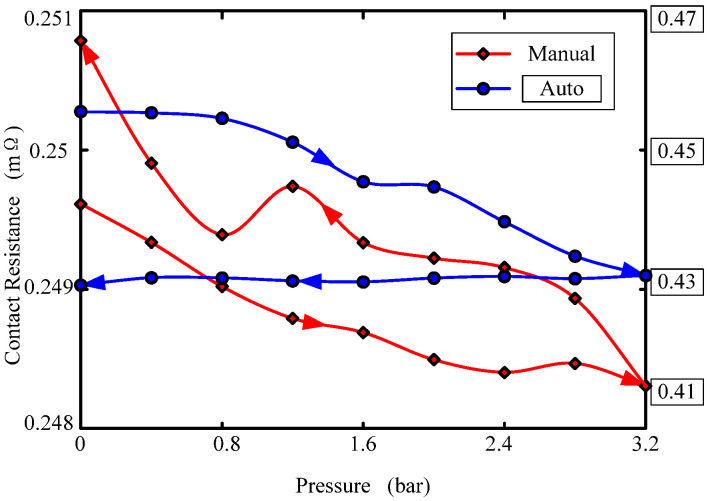
Contact resistance variations with pressure.

Figure 6 shows the variation in static contact resistance under different pressures. The fluid ring is 3#: Cu-Cu. In the figure, the blue line is the contact resistance change in automatic vacuum perfusion, the red line is the contact resistance change in manual perfusion at atmospheric pressure, and the arrow direction indicates the direction of pressure change. As can be seen from the figure: (1) with the increase in pressure, the contact resistance of the fluid ring gradually decreases; (2) the contact resistance of atmospheric manual perfusion fluid ring increases with the decrease in pressure, and the contact resistance of the vacuum automatic perfusion fluid ring is virtually constant with the decrease in pressure. The reason is that the conductive fluid has large surface tension, and it is easy to form a meniscus with the rough surface of the metal electrode, which cannot completely wet the rough solid surface. Increasing the contact pressure can increase the wettability of the rough surface, make the contact more complete, increase the contact area, and decrease the contact resistance. The residual gas on the rough surface of the manually perfused fluid ring electrode is more than that of the vacuum perfused electrode, and the gas deforms under the action of pressure, so that the contact area between the conductive fluid and the metal electrode surface changes little, so the contact resistance changes little. There is less residual gas on the rough surface of the vacuum-perfused fluid ring. Under the action of pressure, the contact area between the conductive fluid and the metal electrode surface significantly changes, and the contact resistance significantly changes.

**Figure 7:**
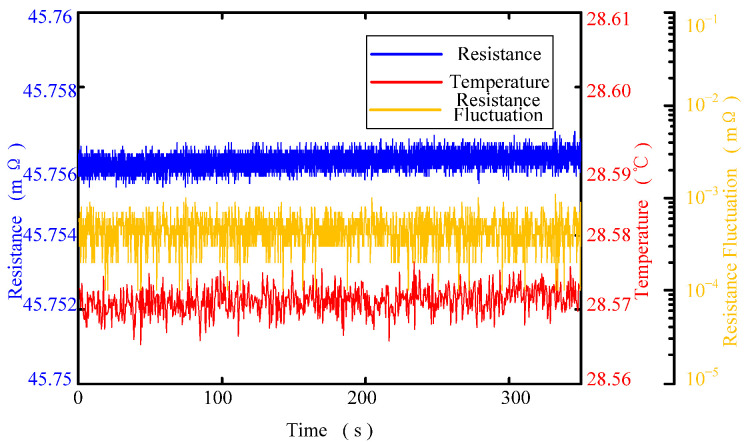
Static contact resistance and resistance fluctuation at 28.57 °C.

**Figure 8:**
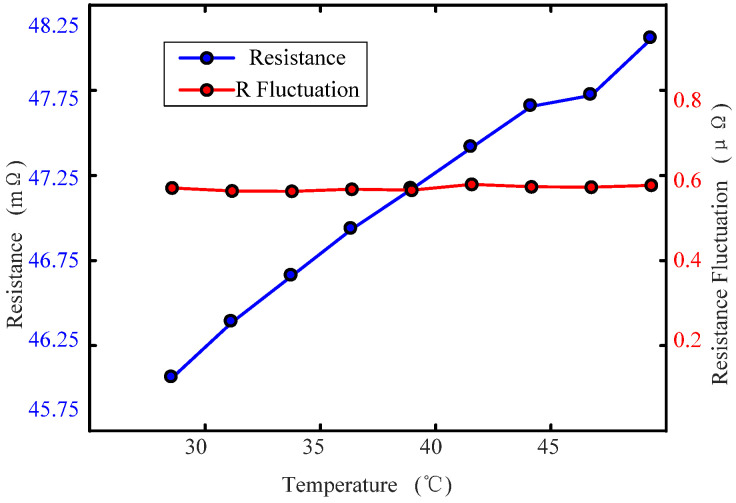
Static contact resistance and resistance fluctuation variations with temperature.

Figure 7 and Figure 8 show the variation in static contact resistance of the fluid ring, which is 3#: Cu-Cu, at different temperatures. Figure 7 shows the variation curve of the contact resistance and its fluctuation curve when the temperature is 28.57 °C. Figure 8 shows the variation curve of the static resistance of the fluid ring and its fluctuation with temperature. It can be seen from the figures that: (1) during a test period of contact resistance, the ambient temperature changes little and the temperature fluctuation is less than 0.02 °C, and so it can be considered that the ambient temperature is constant during the test; (2) the static contact resistance of the fluid ring has a slight fluctuation, and the fluctuation range is between 0.1~1 μΩ and the main concentration area is 0.3~0.7 μΩ. (3) With the increase of temperature, the total resistance of the fluid ring increases; (4) within the range of temperature variation, the fluctuation of the contact resistance of the fluid ring remains unchanged.

**Figure 9:**
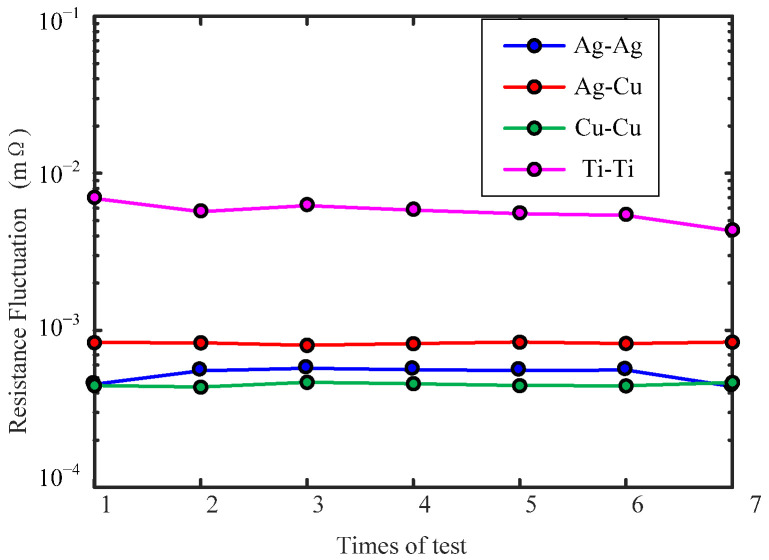
Contact resistance fluctuation curves of different electrode materials.

Figure 9 shows the comparison of contact resistance fluctuation curves of fluid rings with different electrode materials. The abscissa is the number of static resistance tests, and the ordinate is the contact resistance fluctuation value. It can be seen from the figure that: (1) the resistance fluctuation repeatability of the four materials measured multiple times is better for the fluid ring made of Ag and Cu, with repeatability being less than 5%, while the repeatability of the fluid ring of Ti is as high as 15%. (2) The smallest resistance fluctuation value of the fluid ring is for 3#: Cu-Cu, with an average of 0.451μΩ, followed by 1#: Ag-Ag, with an average of 0.528 μΩ, and 2#: Ag-Cu, with an average of 0.83 μΩ, while the contact resistance fluctuation of 4#: Ti-Ti fluid ring is the largest, with an average value of 5.7 μΩ, which is 12.6 times of that of 1#: Ag-Ag. The reason is that the resistivity of electrode material and the wettability of conductive fluid to Cu, Ag and Ti are different. The resistivity of Ag, Cu and Ti are (0.0162, 0.0175 and 0.42) μΩ·m [27], respectively. The resistivity of Ti is much higher than that of Ag and Cu, so the contact resistance of Ti is much higher than that of Ag and Cu. The static resistance values of the four fluid rings in the figure are 46.01 mΩ, 45.65 mΩ, 45.78 mΩ and 102.76 mΩ, corresponding to 1#–4#, respectively. There is less difference between the contact resistance of Cu and Ag electrode materials and conductive fluid. At the same time, the static contact angles of conductive fluid to Cu and Ag at room temperature are 130° and 132.5°, and the static contact angles of Ti are 155°. The wettability of conductive fluid to Ti is worse, and the contact resistance is much larger than that of Ag and Cu.

**Figure 10:**
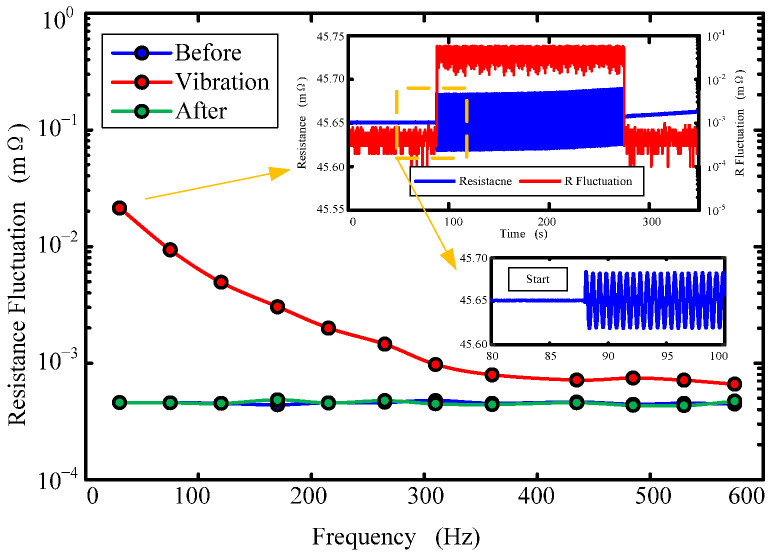
Contact resistance fluctuation variations with frequency.

Figure 10 shows the variation of contact resistance fluctuation with frequency when the input frequency of the angular shaker is 25 Hz and the input voltage is 5 V. The fluid ring is 3#: Cu-Cu. As can be seen from the figure, the fluctuation of contact resistance decreases with the increase in frequency. According to Equation (15), the contact resistance fluctuation is inversely proportional to the square of the input frequency of the high-frequency shaking table, and the contact resistance fluctuation before and after vibration remains basically unchanged. With the increase in frequency, the amplitude of contact resistance fluctuation gradually decreases. It also can be seen that, when the high-frequency shaking table starts, the contact resistance value of the fluid ring starts to regularly oscillate with the angular vibration. When the high frequency shaking table stops vibrating, the oscillation of the fluid ring contact resistance stops. By comparing the maximum amplitude of contact resistance of the fluid ring during vibration and before and after vibration, the contact resistance value during vibration is obviously nearly two orders of magnitude higher than that before and after vibration. In Equation (15), during the process of high-frequency shaking table vibration, the conductive fluid moves in the fluid ring, which changes the contact area between the conductive fluid and the electrode surface, leading to changes in the contact resistance.

**Figure 11:**
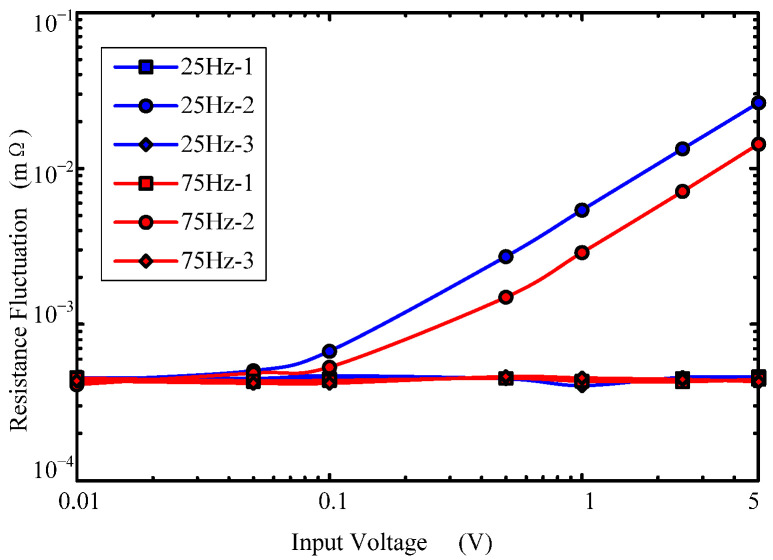
Contact resistance fluctuation variations with angular vibration amplitude.

Figure 11 shows the characteristics of dynamic contact resistance fluctuations with input voltage amplitude. The figure shows the fluctuation in contact resistance when the input voltage amplitude is 5 V, and the frequency is 25 Hz and 75 Hz. In the figure, −1, −2 and −3 represent the contact resistances before vibration, during vibration and after vibration, respectively. As can be seen: (1) when the input voltage *U_r_* is greater than 0.1 V, the fluctuation of contact resistance is linearly related to the input voltage amplitude; (2) when the input voltage *U_r_* is less than 0.05 V, the resistance fluctuation during vibration is small, which is close to the resistance fluctuation before and after vibration. If the input voltage amplitude continues to be reduced, the contact resistance fluctuation will not accurately reflect the angular vibration.

**Figure 12:**
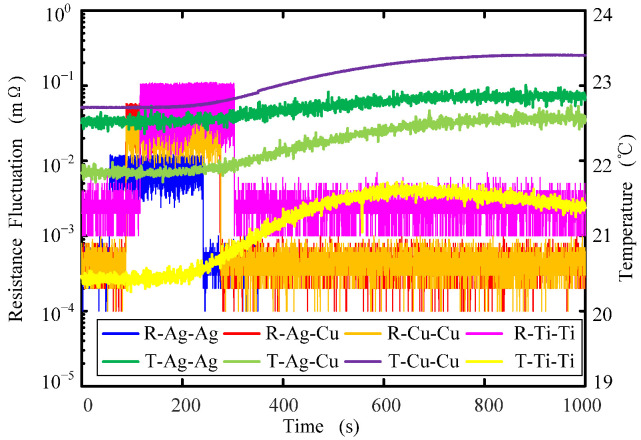
Dynamic contact resistance changes in fluid rings of electrodes made from different materials.

Figure 12 shows the dynamic resistance changes of fluid rings made of different materials at 25 Hz frequency and 5 V input voltage amplitude. It can be seen from the figure that: (1) under the same angular vibration input, the dynamic resistance fluctuation of electrode materials 1#: Ag-Ag is the smallest, followed by 3#: Cu-Cu and 2#: Ag-Cu, and the largest fluctuation is the 4#: Ti-Ti fluid ring; (2) the fluctuation in dynamic contact resistance is similar to that of static resistance, which is related to the resistivity of the metal electrode and the wettability of conductive fluid on the electrode surface.

**Figure 13:**
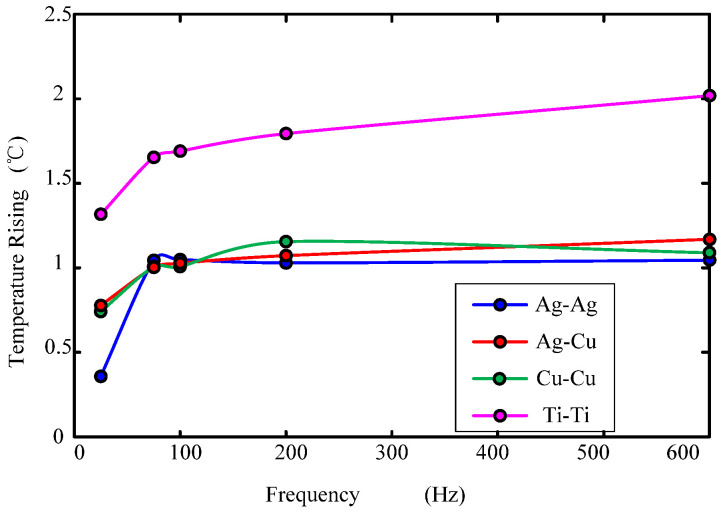
Temperature variations with frequency.

Figure 13 shows the temperature rise curve of the fluid ring when the input voltage amplitude of the high-frequency shaking table is 5 V. As can be seen from the figure: (1) the temperature rising of the fluid ring increases with the increase of the angular vibration frequency; (2) the electrode material for the fluid ring temperature rise of Ti was obviously higher than that of the rest of the electrode material. This was due to the low thermal conductivity of Ti, 17 W/(mK), which is only 1/22.5 of copper, leading to surface friction heat conductive fluid that is not dispersed in time, so as to make the electrode temperature rise larger. This is the same for Cu and Ag, due to their high thermal conductivity and fast heat dissipation, so the electrode temperature rise is smaller. An increase in the temperature will increase contact resistance. In Figure 10 and Figure 12, the reason why the contact resistance value is larger after the vibration than before the vibration is that the temperature rise during the vibration process is caused by the temperature rise.

**Figure 14:**
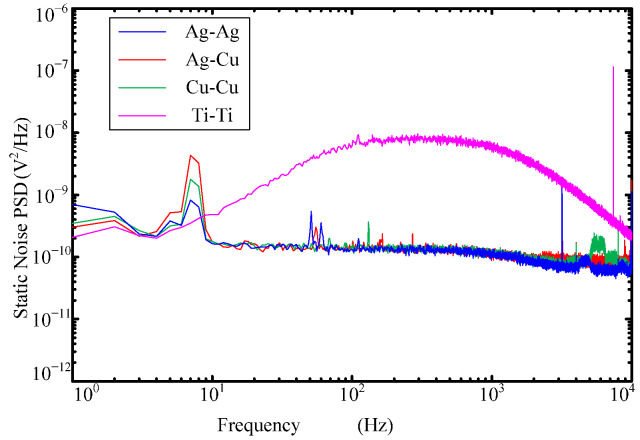
Static noise power spectral density curves of MHD sensors with different material electrode fluid rings.

**Figure 15:**
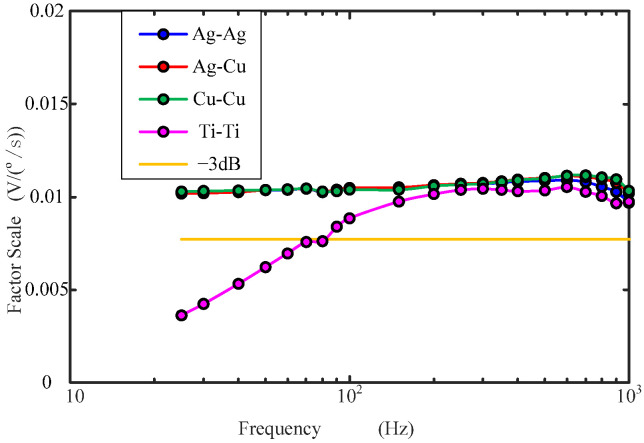
Factor Scale of MHD sensors with different material electrode fluid rings.

Figure 14 and Figure 15 show the static noise power spectral density (PSD) and Factor Scale curves of the MHD sensors with different metal electrode fluid rings. It can be seen from the figures that the static noise PSD curve of the MHD sensor with 1#: Ag-Ag, 2#: Ag-Cu and 3#: Cu-Cu fluid rings is flat in the high frequency band of 10Hz–1000Hz, except for the low frequency < 10Hz. However, the static noise PSD curve of the 4#: Ti-Ti fluid ring increases with the increase in frequency in the range of 10Hz–1000Hz, and there is no flat segment in the frequency band range of the MHD sensor, which leads to its performance deterioration. The Factor Scale of 1#: Ag-Ag, 2#: Ag-Cu and 3#: Cu-Cu MHD sensors have good flatness in the range of 25Hz–1kHz, and are all above the −3dB line, while the scaling factor curve of 4#: Ti-Ti is below the −3dB line when it is below 80Hz. Therefore, the Factor Scale performance of 4#: Ti-Ti sensor is worse than that of the other three. It can be concluded that reducing the contact resistance of the fluid ring is beneficial to improve the performance of the MHD sensor.

## 6. Conclusions

This paper analyzed the influencing factors of the contact resistance between conductive fluid and metal electrode in the fluid ring of the MHD sensor, and an experimental study on the static and dynamic contact resistance between different metal electrodes of Ag, Cu, Ti and conductive fluid. The following conclusions were drawn:

(1) The static contact resistance between the conductive fluid and the metal electrode decreases with the increase of pressure, and increases with the increase of temperature.

(2) Static and dynamic contact resistances of different materials are related to the resistivity of the material and the wettability between them. The lower the resistivity of the material, the better the wettability of the conductive fluid to the metal electrode, and the smaller the contact resistance.

(3) The contact resistance of the fluid ring oscillates with angular vibration, and the amplitude of contact resistance fluctuation is proportional to the amplitude of angular vibration input voltage, and is inversely proportional to the square of angular vibration frequency.

(4) The contact resistance has a great influence on the static noise PSD curve and frequency response characteristic curve of the MHD sensor, and the MHD sensor with low contact resistance has a better performance.

In conclusion, by selecting electrode materials with low resistivity and good wettability, or improving the wettability between conductive fluid and electrode materials, the fluid ring contact resistance can be reduced and the performance of MHD sensor can be improved.
